# Lipid nanoparticles that deliver IL-12 messenger RNA suppress tumorigenesis in MYC oncogene-driven hepatocellular carcinoma

**DOI:** 10.1186/s40425-018-0431-x

**Published:** 2018-11-20

**Authors:** Ian Lai, Srividya Swaminathan, Virginie Baylot, Adriane Mosley, Renumathy Dhanasekaran, Meital Gabay, Dean W. Felsher

**Affiliations:** 1 Division of Medical Oncology, Departments of Medicine and Pathology, Stanford University, CA, Stanford, USA; 2 Division of Gastroenterology and Hepatology, Stanford University, Stanford, CA USA

**Keywords:** HCC, IL-12, Immunotherapy

## Abstract

Interleukin-12 (IL-12) is a promising candidate for cancer immunotherapy because of its ability to activate a number of host immune subsets that recognize and destroy cancer cells. We found that human hepatocellular carcinoma (HCC) patients with higher than median levels of IL-12 have significantly favorable clinical outcomes. Here, we report that a messenger RNA (mRNA) lipid nanoparticle delivering IL-12 (IL-12-LNP) slows down the progression of MYC oncogene-driven HCC. IL-12-LNP was well distributed within the HCC tumor and was not associated with significant animal toxicity. Treatment with IL-12-LNP significantly reduced liver tumor burden measured by dynamic magnetic resonance imaging (MRI), and increased survival of MYC-induced HCC transgenic mice in comparison to control mice. Importantly, IL-12-LNP exhibited no effect on transgenic MYC levels confirming that its therapeutic efficacy was not related to the downregulation of a driver oncogene. IL-12-LNP elicited marked infiltration of activated CD44^+^ CD3^+^ CD4^+^ T helper cells into the tumor, and increased the production of Interferon γ (IFNγ). Collectively, our findings suggest that IL-12-LNP administration may be an effective immunotherapy against HCC.

**Electronic supplementary material:**

The online version of this article (10.1186/s40425-018-0431-x) contains supplementary material, which is available to authorized users.

## Introduction

Cytokines are crucial signaling mediators that alert the immune system to the presence of foreign antigens. Cytokine treatment may also be an effective immune therapy for the treatment of cancer. Interleukin-12 (IL-12) has anti-tumor activity in a variety of preclinical models [1–6]. The anti-tumor activity of IL-12 can be attributed to its ability to bridge innate and adaptive immune surveillance mechanisms, thereby creating a long-lasting immune response against cancers [7].

IL-12 was originally identified as “natural killer (NK) cell stimulatory factor” [8], but has subsequently been shown to exert effects on other immune compartments such as NK T cells, T-lymphocytes [9], cells of myeloid origin [10], and tonsillar B cells [11]. Despite its pleiotropic effects on multiple immune compartments, IL-12 is particularly important during T cell activation initiated by T cell receptor (TCR) signaling [12, 13]. TCR activation promotes sensing of IL-12 by the induction of IL-12 receptors (IL-12R), and subsequently leads to secretion of Interferon-γ (IFNγ) by the activated T cells [14]. IFNγ can kill tumor cells directly or by recruiting and activating key immune subsets such as macrophages and NK cells that recognize tumor cells [15]. Thus, IL-12 is a potent anti-cancer agent directly and indirectly through the recruitment and activation of other immune effectors, and secretion of cytokines.

Administration of IL-12 has been previously demonstrated to have therapeutic benefits in multiple preclinical models of cancer, such as breast [16], liver [17, 18], and colon [19]. However, the lack of intravenous approaches to robustly deliver IL-12 to the tumor and avoid cytotoxicity of the surrounding normal tissues has slowed the development of IL-12 as a modality to treat human cancers [20].

Amongst solid tumors, treatment of hepatocellular carcinoma (HCC) remains a significant clinical challenge [21–24]. Hence, in the current study, we examined the efficacy of a novel mRNA lipid nanoparticle based approach for the in situ delivery and production of IL-12 (IL-12-LNP) in suppressing tumor progression in a primary transgenic mouse model of refractory MYC-driven hepatocellular carcinoma (HCC, [25]). Overexpression of the MYC oncogene is a common feature of aggressive HCCs [26–29]; making our model particularly relevant for pre-clinical studies involving novel therapeutic agents, such as IL-12-LNP.

While molecular targeting of MYC would represent the most ideal treatment strategy for combating MYC-driven HCCs [30–33], MYC inhibitors that do not exert toxic effects on normal tissues are yet to be identified. By selecting our model of MYC-driven HCC, we hoped to identify if IL-12-LNP can be an effective non-toxic alternative to MYC inhibition for treating aggressive HCCs. We demonstrate that IL-12-LNP is well distributed to the liver tumor and surrounding normal liver tissue, not associated with animal and liver toxicity, suppresses tumor growth, and increases survival of our transgenic mice predisposed to developing MYC-driven HCC.

## Methods

### Human HCC patient data mining and analysis

Gene expression data were analyzed from a previously published retrospective study conducted on 387 paraffin-embedded liver tissues obtained from HCC patients (Gene Expression Omnibus (GEO) Accession Number GSE10143) [34]. Normalized mRNA expression data and clinical data (survival times and outcomes) of patients (*n* = 387) were obtained from PRECOG (Prediction of Clinical Outcomes from Genomic Profiles, https://precog.stanford.edu/index.php). Patients (*n* = 144) for whom survival data was available were separated into two groups based on the median levels of IL12A mRNA, as IL12A^High^ and IL12A^Low^. Kaplan-Meier Survival Analysis was then used to compare the overall survival (OS) probabilities between the two groups to determine if IL12A mRNA levels in liver tissue can serve as an independent predictor of clinical outcome in HCC patients.

### HCC transgenic mice

**Fig. 1:**
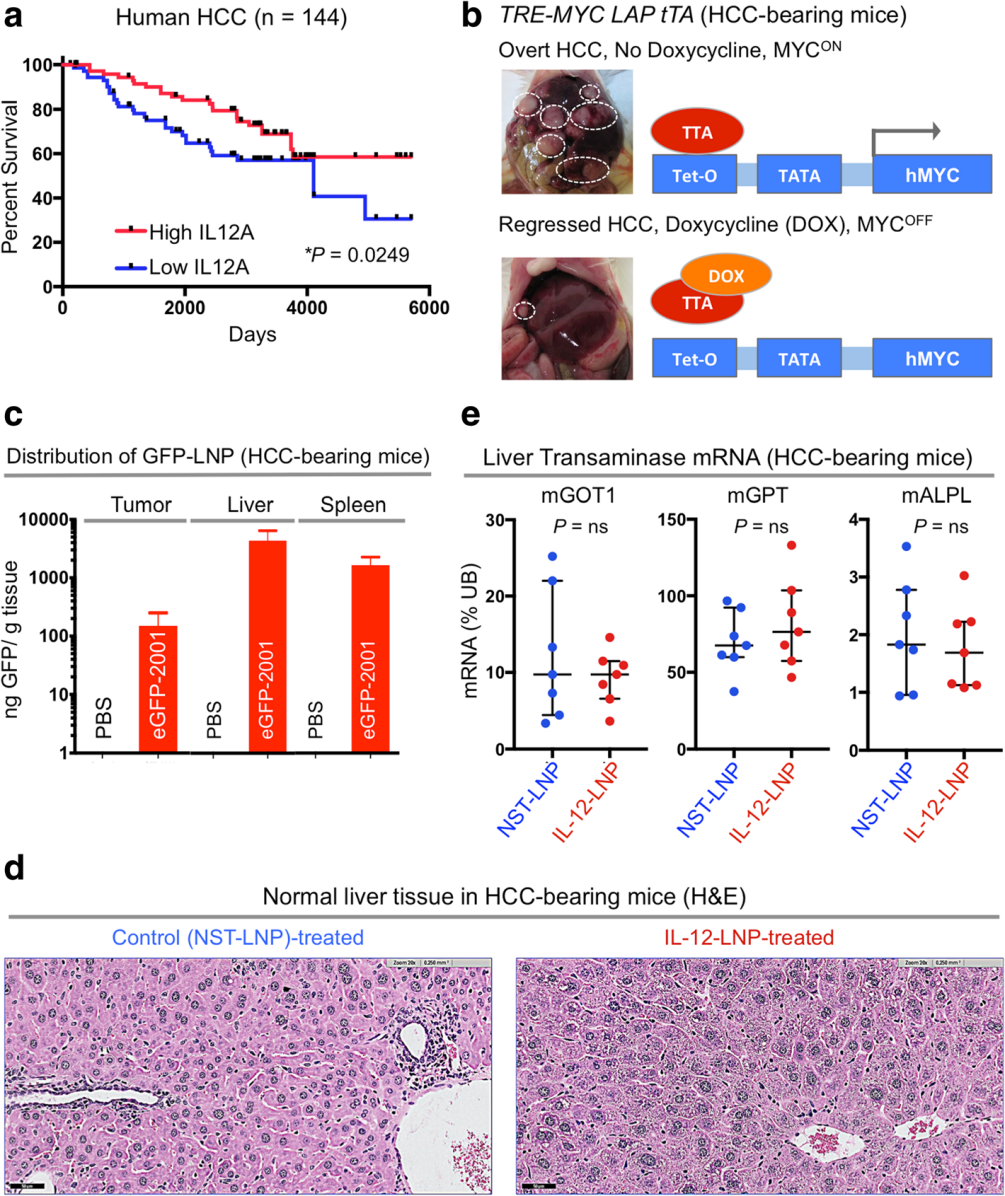
IL-12-LNP as an effective and non-toxic treatment for hepatocellular carcinoma. **a** Kaplan-Meier survival analysis comparing overall survival (OS) probabilities of HCC patients (*n* = 144) divided into two groups (High IL-12 and Low IL-12) based on their median expression of IL-12. *P*-value was calculated using the log-rank test. **b** Morphology of livers (left) and diagrammatic representation (right) of the transgenic mouse model of MYC-driven HCC used in the study, before and after MYC inactivation. White dotted circles represent examples of tumor nodules in the liver. **c** Biodelivery of IL-12 oligonucleotide therapy (IL-12-LNP) and corresponding controls (NST-LNP) designed by Onkaido, in HCC (tumor, left), whole liver (middle), and spleen (right). **d** Hematoxylin & Eosin (H&E) analysis of normal liver tissue surrounding tumors isolated from control (NST-LNP, *n* = 7) and IL-12 treated (IL-12-LNP, n = 7) HCC-bearing mice. One representative image is shown from each group of mice. Scale bars = 50 μm. **e** Quantitative real time PCR comparing mRNA levels of liver transaminases between NST-LNP (n = 7) and IL-12-LNP (n = 7) treated HCC-bearing mice. Each dot represents a single mouse and is the average of three technical replicates. Data are represented as median ± interquartile range. *P*-values were calculated using Student’s t-test. *P* values: ns = not significant, * *p* < 0.05, ** *p* < 0.01, *** *p* < 0.001, **** *p* < 0.0001

All animals were housed in a pathogen-free environment at Stanford University and all procedures were performed in accordance with Stanford’s Administrative Panel on Laboratory Animal Care (APLAC) protocols. *LAP*-*tTA/tet-O-hMYC* transgenic lines, as previously described [25] (Fig. 1b, Model), were administered weekly doses of 0.1 mg/mL doxycycline (Sigma) in drinking water during mating (in utero) up until 4 weeks of age. Mice were screened for tumors under 50 mm^3^, developing at approximately 2–3 months of age, with euthanasia occurring following completion of treatment or when tumor burden exceeded 5,000 mm^3^.

### Magnetic Resonance Imaging (MRI)

MRI was performed weekly using a 7 T small animal MRI (Brucker) at the Stanford Small Animal Imaging Facility as previously described [35]. Briefly, animals were anesthetized with 1–3% isofluorane and fed into the MRI scanner containing a 40 mm Varian Millipede RF coil (ExtendMR LLC). Tumor volumes were quantified from acquired DICOM images using Osirix image processing software (Osirix).

### Lipid Nanoparticle (LNP) administration

Modified mRNA LNPs containing IL-12 or a control oligonucleotide containing no start codon (NST-LNP) were synthesized and provided by Onkaido (Moderna Therapeutics). Upon tumor detection, mice were administered weekly intravenous doses of the NST-LNP (0.025 mg/kg) or IL-12-LNP (0.025 mg/kg), for 3 weeks (20 mice per group) or up to 9 weeks for the determination of a survival curve (14–15 mice per group).

### Immunohistochemistry

Tissues were fixed in 10% paraformaldehyde and embedded in paraffin for sectioning. Sections were deparaffinized and stained with c-MYC (1:150, Clone #EP121, monoclonal, Epitomics), CD4 (1:100, Clone #EPR19514, monoclonal, Abcam), CD3 (1:100, polyclonal, Abcam), and CD44 (1:2000, polyclonal, Abcam) overnight, and incubated with biotinylated anti-mouse IgG for 30 min at room temperature (1:300 Vectastain ABC kit, Vector Labs). Sections were developed using 3,3′- Diaminobenzidine (DAB), counterstained with hematoxylin, and mounted with permount. Stained sections were scanned and imaged with a Digital Pathology Slide Scanner (Philips). Representative tumor tissue sections were quantified from acquired images using ImageJ software (NIH).

### Quantitative RT-PCR

Quantitative real-time PCR carried out with the SYBRGreenER mix from Invitrogen according to standard PCR conditions and an ABI7900HT real-time PCR system (Applied Biosystems). Primers for quantitative RT-PCR are listed below:

mGOT1_F 5’-AGAGAAAGATGCGTGGGCTA-3’.

mGOT1_R 5’-TGGACCAGGTGATTCGTACA-3′.

mGPT_F 5’-AAGGCTAAACTCACGGAGCA-3’.

mGPT_R 5’-CTCTTCCAGGAGGCACAGAC-3′.

mALPL_F 5’-GCTGATCATTCCCACGTTTT-3’.

mALPL_R 5’-CTGGGCCTGGTAGTTGTTGT-3′.

mIFNG_F 5’-ACTGGCAAAAGGATGGTGAC-3’.

mIFNG_R 5’-TGAGCTCATTGAATGCTTGG-3’.

### Statistical analysis

Results (Mean ± SEM) were analyzed for statistical significance by Student’s t-test and one-way ANOVA, using Prism (Graphpad Software, Inc.). The Kaplan-Meier estimate was used for survival analysis. Log-rank test was used to calculate statistical significance for all survival studies. The threshold for statistical significance was set at *P* < 0.05. *P* values: ns = not significant, * *p* < 0.05, *** *p* < 0.001, **** *p* < 0.0001.

## Results

### IL-12 expression predicts survival of humans with HCC

Gene expression profiles of surrounding normal liver tissues have been previously demonstrated to predict the prognosis of patients with hepatocellular carcinoma (HCC, GSE10143) [34]. We examined if the expression of IL-12 mRNA in the surrounding normal liver tissue of human HCC patients can predict clinical outcome. A cohort of 144 HCC patients (GSE10143) were divided into two groups based on median expression of IL-12 mRNA levels [34] (Fig. 1a). Patients with higher than median IL-12 mRNA levels in normal liver tissue surrounding the HCC had a significantly prolonged overall survival (OS) than patients with lower than median IL-12 levels (Fig. 1a).

Since IL-12 is a favorable prognostic factor for HCC, we hypothesized that HCC may respond to IL-12-based therapies. We evaluated the efficacy of a novel IL-12 mRNA therapy in our primary tetracycline (tet)-inducible transgenic mouse model of MYC oncogene-driven HCC [25] (Fig. 1b). In this transgenic model, full-blown HCC tumors completely regress only upon MYC inactivation (Fig. 1b). We investigated whether IL-12 mRNA-based therapy can delay MYC-driven HCC progression, and therefore serve an alternative to MYC inhibition for treating HCCs.

### A non-toxic IL-12 mRNA-based immunotherapy for HCC

Nucleic acids, mRNA in particular, are beginning to be widely employed in cancer immunotherapy [36–40]. Here, we use a modified IL-12 mRNA encapsulated within a lipid nanoparticle (LNP), referred as “IL-12-LNP”. The IL-12 mRNA was engineered to have the desired sequence and modifications for producing a functionally active IL-12 protein. Once within the organism, the LNP releases the IL-12 mRNA into cells where it is decoded by the ribosomal machinery into IL-12 protein. A control oligonucleotide with no start codon (NST) encapsulated in LNPs (referred here as “NST-LNP” controls) was used as a vehicle control. Both the IL-12-LNP and NST-LNP were delivered intravenously.

First we measured the biodistribution of intravenously administered LNPs carrying a GFP tagged NST mRNA cargo in our transgenic mouse model of MYC-driven HCC. NST-GFP-LNP was efficiently delivered to the tumor (HCC, Fig. 1c, left), non-malignant regions of the liver (Fig. 1c, middle) and spleen (Fig. 1c, right) of the mice, suggesting its superiority over existing intratumoral methods of IL-12 delivery [20].

Next, we evaluated whether IL-12-LNP treatment induces animal toxicity. The administration of IL-12-LNP did not significantly alter the body weights of healthy mice as compared to NST-LNP-treated controls (Additional file 1a). We found that normal tissue surrounding liver tumors in HCC-bearing mice show no evidence of increased hepatitis post IL-12-LNP treatment in comparison to NST-LNP-treated controls (Fig. 1d). To definitively rule out liver toxicity because of IL-12-LNP treatment, we also compared the mRNA levels of common liver transaminases (Glutamic-Oxaloacetic Transaminase 1, GOT1; Alkaline Phosphatase, ALPL; Glutamic Pyruvic Transaminase, GPT) in normal liver tissue between NST-LNP and IL-12-LNP treated HCC-bearing mice (Fig. 1e). We observed that IL-12-LNP treatment does not significantly stimulate the production of liver transaminases (Fig. 1e). Based on these findings, we conclude that IL-12-LNP therapy does not induce liver toxicity.

### Short-term administration of IL-12-LNP significantly reduces HCC tumor burden

**Fig. 2:**
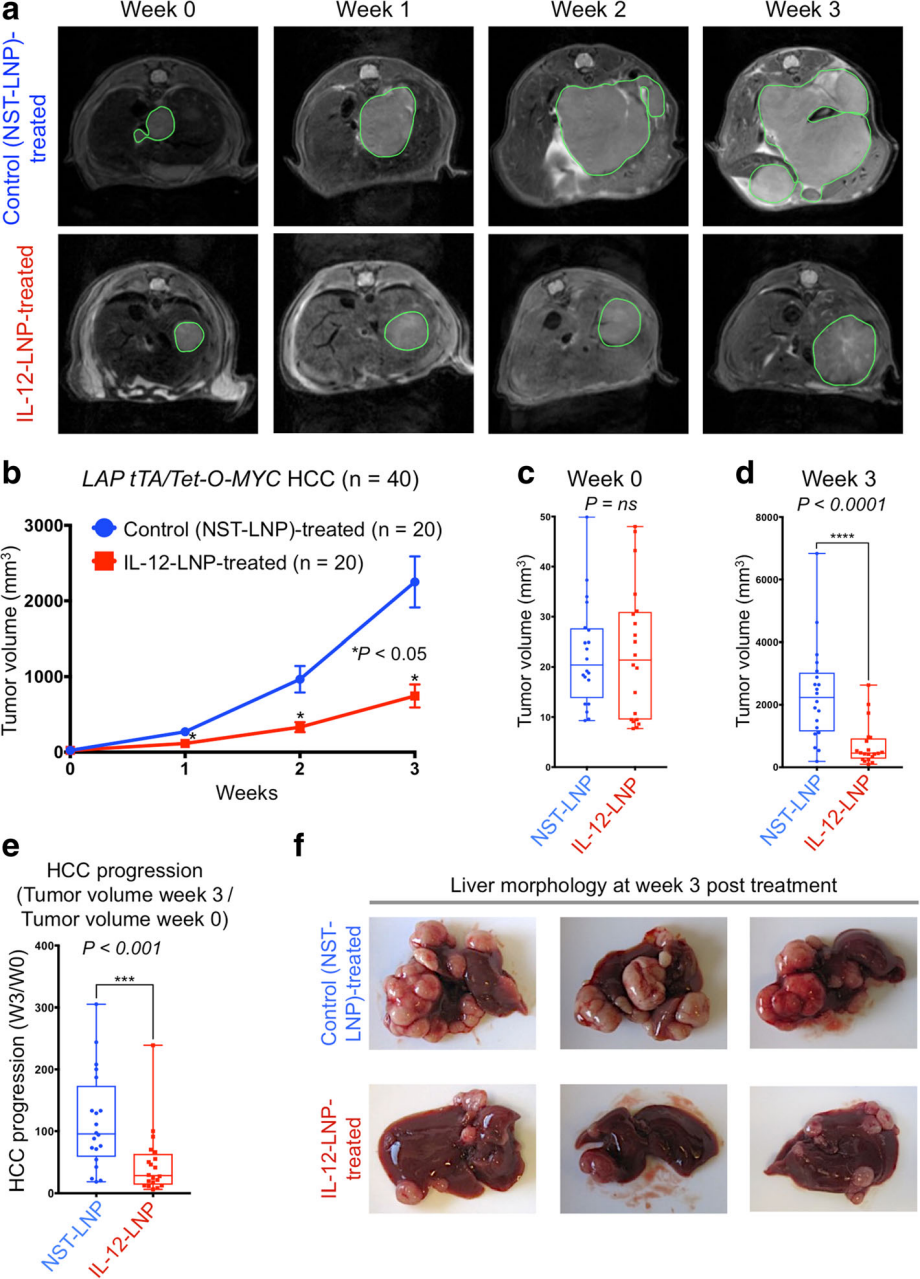
Short-term treatment with IL-12-LNP significantly slows down HCC progression. **a** Serial magnetic resonance imaging (MRI) of primary transgenic mice bearing MYC-driven HCC that were either administered control NST-LNP (*n* = 20) or IL-12-LNP (n = 20) intravenously at weeks 0, 1, 2 and 3 post treatment. Representative images of one mouse in each group are shown for the indicated time points. **b** Comparison of HCC progression (tumor burden) as computed by MRI after treatment with control (NST-LNP, n = 20) or IL-12 (IL-12-LNP, n = 20) oligonucleotides for three weeks. Tumor burden for each time point represented as mean ± SEM. **c** Box plots depicting absolute HCC burden in individual mice before starting treatment with IL-12-LNP (n = 20) or NST-LNP (n = 20) controls (week 0), as computed by MRI. **d** Box plots depicting absolute HCC burden in individual mice at the end of short-term treatment with IL-12-LNP (n = 20) or NST-LNP (n = 20) controls (week 3), as computed by MRI. **e** Box plots depicting fold change in HCC burden of individual mice at week 3 with respect to initial tumor burden before beginning treatment (week 0) between NST-LNP and IL-12-LNP treated groups. P-values were calculated using Student’s t-test. P values: ns = not significant, * p < 0.05, *** p < 0.001, **** *p* < 0.0001. **f** Morphology of livers isolated from control (NST-LNP, n = 20) and IL-12-treated (IL-12-LNP, n = 20) HCC mice at week 3 post treatment. Three representative images are shown from each group

The effects of short-term (3 week) treatment with IL-12-LNP (*n* = 20) versus NST-LNP controls (n = 20) on tumor progression in a transgenic mouse model of MYC-driven HCC [25] were measured by magnetic resonance imaging (MRI). MRI is a sensitive methodology to robustly visualize the dynamic process of hepatocellular carcinogenesis over a given period of time. The short-term IL-12-LNP administration significantly reduced liver tumor burden and delayed tumor progression when compared to NST-LNP-treated controls (Fig. 2a-b).

HCC transgenic mice in both groups (control and IL-12-treated) had comparable tumor burdens (0–50 mm^3^) at the start of treatment (week 0, Fig. 2c). At week 3, we observed a significant reduction in the absolute tumor burden in the IL-12-treatment group (Fig. 2d). HCC progression was quantified as a ratio of the change in tumor burden at week 3 with respect to week 0 for individual mice in each group (Fig. 2e). HCC progression was significantly delayed in IL-12-treatment cohort as compared to the NST-LNP-treated control group (Fig. 2e). Concordant with the reduced tumor growth measured by MRI, there was a reduction in the gross number of tumor nodules in liver, in mice sacrificed three weeks after IL-12-LNP administration (Fig. 2f). Hence, IL-12-LNP treatment significantly reduces HCC tumor growth and delays tumor progression as demonstrated by dynamic MRI.

### Treatment with IL-12-LNP confers a significant survival advantage in transgenic mice

**Fig. 3:**
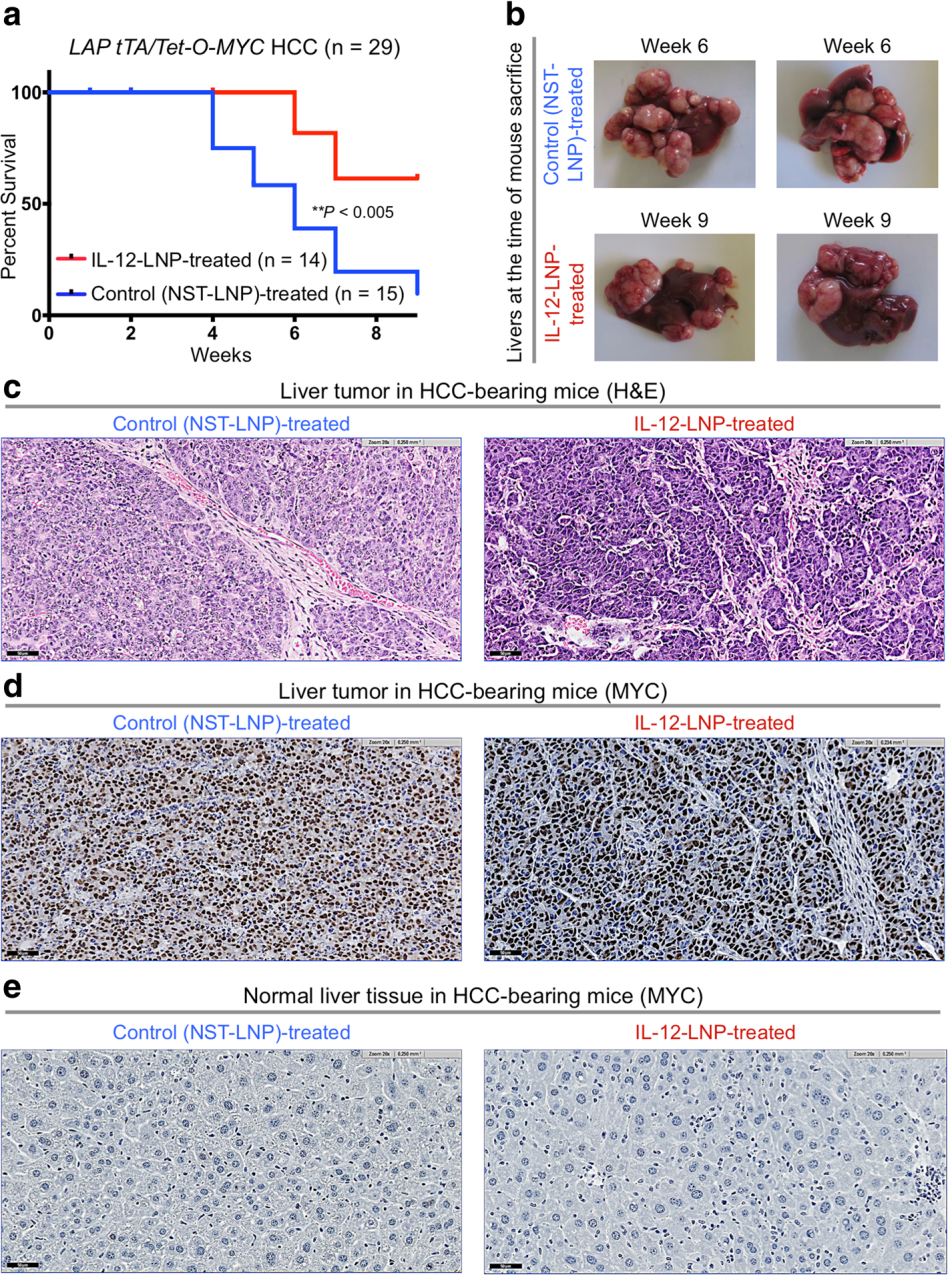
Treatment with IL-12-LNP confers a significant survival advantage on HCC-bearing mice independently of MYC. **a** Kaplan-Meier survival analysis comparing overall survival (OS) between control (NST-LNP, *n* = 15), and IL-12-treated (IL-12-LNP, n = 14) MYC-driven HCC mice. P-values were calculated using log-rank test. P values: ** *p* < 0.01. **b** Morphology of livers isolated from control (NST-LNP, n = 15) and IL-12-treated (IL-12-LNP, n = 14) HCC mice at the end of treatment schedule. Two representative images are shown from each group of mice. **c** Hematoxylin & Eosin (H&E) analysis of liver tumors isolated from control (NST-LNP, n = 7) and IL-12 treated (IL-12-LNP, n = 7) HCC-bearing mice at the end of treatment. **d-e** Immunohistochemistry for transgenic hMYC protein in liver tumor (**d**), and surrounding normal liver (**e**) tissues isolated from control (NST-LNP, n = 7) and IL-12 treated (IL-12-LNP, n = 7) MYC-driven HCC mice. One representative image is shown from each group of mice. Scale bars = 50 μm

The effects of long-term administration of IL-12-LNP were examined in MYC-driven HCC transgenic mice. Two cohorts of HCC-bearing mice (0–50 mm^3^) were each given IL-12-LNP (*n* = 14) or control (NST-LNP, *n* = 15) until the mice were moribund with tumor burden (Fig. 3). IL-12-LNP administration significantly delayed tumor progression and prolonged overall survival of mice in comparison to NST-LNP controls (Fig. 3a). The long-term administration of IL-12-LNP reduced the disease burden as measured by the gross number and size of tumor nodules (Fig. 3b). Hence, IL-12-LNP is therapeutically effective in the treatment of transgenic mice with MYC-driven HCC.

Hematoxylin and Eosin (H&E) staining was performed on livers from IL-12-LNP versus NST-LNP treated groups (Figs. 3c-d). Tumor tissues from mice treated with IL-12-LNP but not NST-LNP were associated with decreased number of tumor cells that were replaced with connective tissue (Fig. 3c). These findings suggest that the IL-12-LNP therapeutic can suppress HCC formation.

### IL-12-LNP does not suppress MYC to cause HCC regression

In our transgenic mouse model of MYC-driven HCC, tumors completely regress upon MYC inactivation (Fig. 1b, Model) [25]. Hence, a trivial explanation for the decreased tumor growth in IL-12-LNP treated mice is that the therapy was reducing MYC levels. Immunohistochemistry (IHC) comparing transgenic human MYC (hMYC) protein expression in liver tumor tissues between control (NST-LNP) and IL-12-LNP-treated groups demonstrated no differences in MYC protein levels (Fig. 3d). As expected, normal liver tissues surrounding the tumor from both groups do not express the transgenic hMYC protein (Fig. 3e). Hence, IL-12-LNP does not suppress tumor growth by decreasing MYC expression.

### Treatment with IL-12-LNP causes HCC regression by inducing an anti-tumor immunological response

IL-12-LNP-treated transgenic mice exhibited changes consistent with the induction of an immune response. The administration of IL-12-LNP significantly increased splenic weights of both healthy (FVB/N, Additional file 2a), and HCC-bearing mice (Additional file 2b), consistent with previously observed hematologic effects of IL-12 treatment, such as immune activation [41]. Additionally, we observed that IL-12-LNP treatment of HCC-bearing mice significantly upregulated the production of IFNγ mRNA in the normal liver tissue surrounding the tumor when compared to NST-LNP treated controls (Additional file 2c). Increased splenic volume (Additional file 2a-b) and induction of IFNγ mRNA (Additional file 2c) following IL-12-LNP treatment suggest that IL-12 may potentially induce anti-tumor immunological changes.

**Fig. 4:**
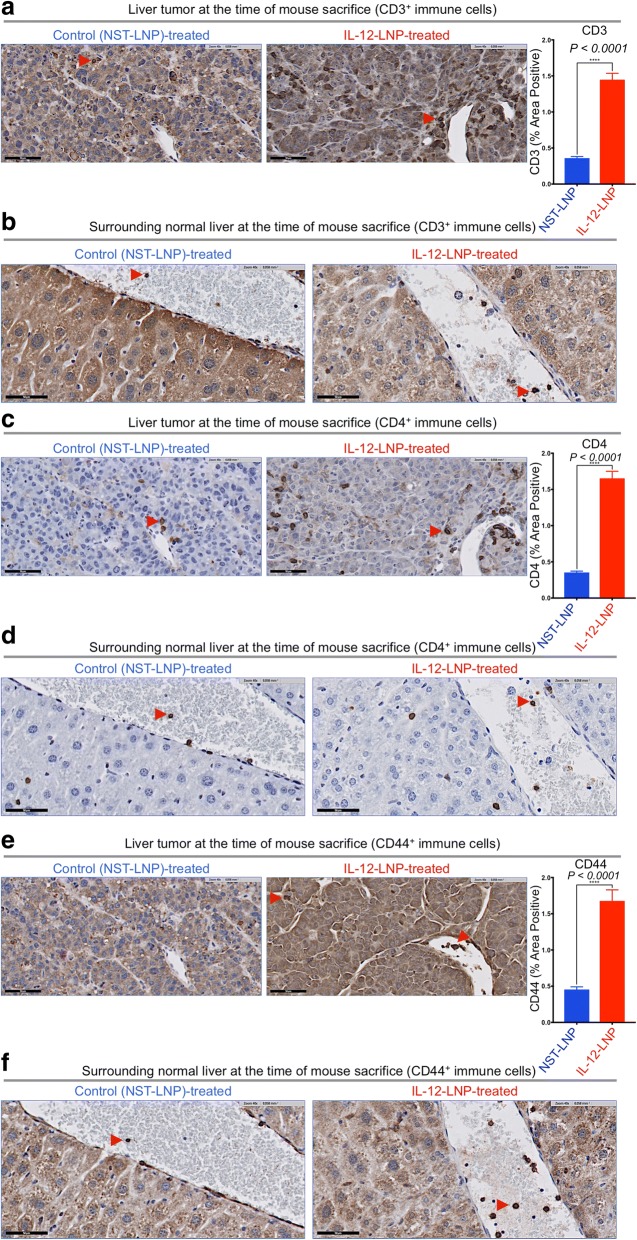
HCC clearance by IL-12-LNP is associated with the recruitment of activated immune cells. **a**-**b** Immunohistochemistry for CD3+ pan T cells in liver tumor (**a**), and surrounding normal liver (**b**) tissues isolated from control (NST-LNP, *n* = 6), and IL-12 treated (IL-12-LNP, *n* = 5) MYC-driven HCC mice. **c**-**d** Immunohistochemistry for CD4+ immune cells (helper T, macrophages and DCs) in the liver tumor (**c**), and surrounding normal liver (**d**) tissues isolated from control (NST-LNP, n = 6), and IL-12 treated (IL-12-LNP, n = 5) MYC-driven HCC mice. **e**-**f** Immunohistochemistry for activated CD44+ T cells in liver tumor (**e**), and surrounding normal liver (**f**) tissues isolated from control (NST-LNP, n = 6), and IL-12 treated (IL-12-LNP, n = 5) MYC-driven HCC mice. Quantification for percent area positive of intratumoral CD3+ (**a**), CD4+ (**c**), and CD44+ (**e**) immune cells is provided to the right of the corresponding IHC images. Quantification is an average of 5 representative IHC images for each group. Red arrowheads within IHC images indicate positive staining for the specific types of immune cells measured. One representative image is shown from each group of mice. Scale bars = 50 μm. P-values were calculated using Student’s t-test. P values: **** p < 0.0001

Next, using IHC, we measured the anti-tumor immunological changes induced by IL-12-LNP treatment. IL-12 has been previously shown to induce anti-tumor immune surveillance through the recruitment of host immune cells such as CD3^+^ CD4^+^ helper T-lymphocytes [7]. Indeed, we found that IL-12-LNP but not NST-LNP-treated mice had increased recruitment of CD3^+^ pan T cells both within the tumor (intratumoral immune cells, Fig. 4a), and in the surrounding normal liver tissue (Fig. 4b). Quantification of representative tumor images confirmed that there was a significant increase of CD3^+^ pan T cell in IL-12-LNP-treated mice (Fig. 4a). Of note, normal liver tissues surrounding the tumor in the IL-12-LNP treated group showed an accumulation of CD3^+^pan T cells around blood vessels (Fig. 4b, right), in contrast to control mice (Fig. 4b, left), suggesting that circulating T cells may be recruited to the tumor site from the vasculature (Fig. 4b).

We performed further IHC-based immunological characterization of the liver tumor tissue and the surrounding normal livers of NST-LNP and IL-12-LNP-treated mice by comparing recruitment of CD4^+^ immune cells (including CD3^+^ T helper, macrophages and dendritic cells (DCs)) between the two groups (Figs. 4c-d). Similar to that observed for CD3^+^ pan T cells, we observed that treatment with IL-12-LNP promoted a robust recruitment of CD4^+^ immune cells both into the tumor and surrounding normal liver tissue when compared to control mice treated with NST-LNP (Figs. 4c-d). Quantification of representative tumor images confirmed that there was a significant increase of CD4^+^ immune cells in IL-12-LNP-treated mice (Fig. 4c).

Our results suggested that the recruitment of a CD3^+^ CD4^+^ helper T cell-mediated immune response (Figs. 4a-d) might in part account for reduced HCC growth after IL-12-LNP-treatment. Hence, we evaluated the activation status of the recruited helper T-lymphocytes post IL-12-LNP treatment by comparing the levels of CD44 (a marker of T cell activation that is expressed by antigen-experienced T cells [42, 43]) between control NST-LNP and IL12-LNP-treated groups (Figs. 4e-f). Quantification of representative tumor images confirmed that there was a significant increase of CD44^+^ immune cells in IL-12-LNP-treated mice (Fig. 4e). IL-12 and TCR stimulation are known to enhance CD44 expression [44]. Of note, we observed an increase in the number of CD44^+^ immune cells recruited to tumor and surrounding normal liver tissue in IL-12-LNP treated mice in comparison to control mice (Figs. 4e-f). CD44 expression on helper CD3^+^ CD4^+^ T cells is known to promote Th1 responses by enhancing the production of IFNγ [45, 46], a phenomenon we also observe in our HCC model (Additional file 2c). We therefore conclude that recruitment of activated CD44^+^ CD3^+^ CD4^+^ T helper lymphocytes into the liver tumor and the surrounding normal liver tissue (Fig. 4) forms an integral part of the anti-HCC immunological response to IL-12-LNP treatment.

## Discussion

**Fig. 5:**
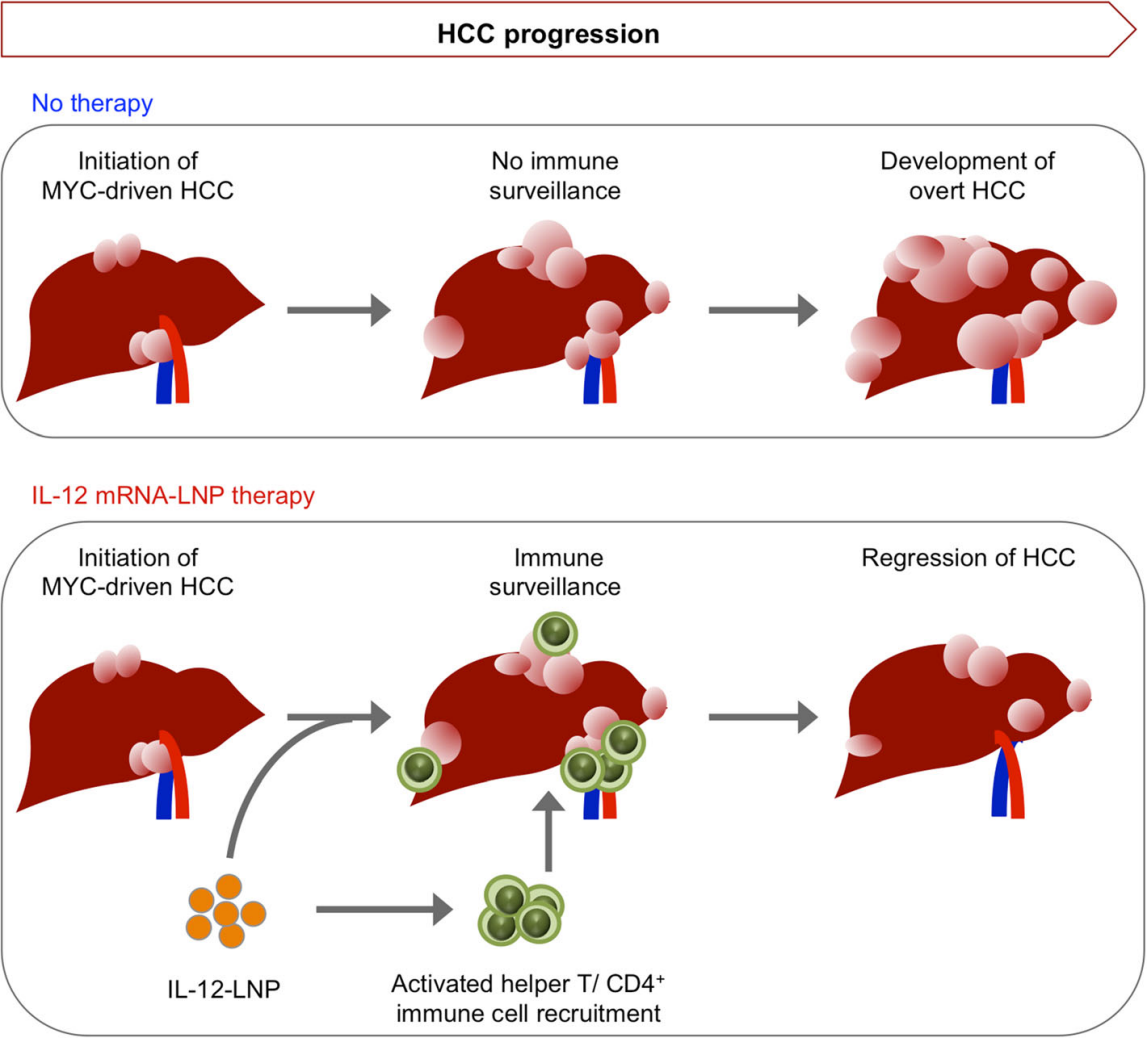
Proposed model by which IL-12-LNP impedes HCC tumor progression. Model depicting the process of HCC progression in the absence (no therapy), and after administration of IL-12-LNP therapy. Intravenous administration of LNPs containing IL-12 mRNA ensures delivery directly to the liver tumor, where it recruits CD44+ CD3+ CD4+ helper T cells and other CD4+ immune cells from the general circulation. CD4+ immune cells then cause HCC regression by raising an immune response to the tumor, and block progression to overt HCC

We demonstrate that lipid nanoparticle based delivery of IL-12 mRNA therapy is effective in suppressing MYC-driven hepatocellular carcinogenesis (Fig. 5, Model). We observed both a reduction in tumor growth through dynamic measurements by MRI and an increase in overall survival in HCC-bearing mice. IL-12-LNP treatment was associated with reduced tumor burden and increased infiltration of activated (CD44^+^, Figs. 4e-f) immune cells such as helper CD3^+^ CD4^+^ T cells into the tumor (Figs. 4a-d). Hence, we have obtained preclinical results suggesting that IL-12-LNP may be an effective immunotherapy against human HCC.

Our study expands upon previous reports suggesting that IL-12 therapy may be an effective treatment for cancer. First, in contrast to previously designed IL-12 therapies, the liposomal IL-12 messenger mRNA therapeutic (IL-12-LNP) does not cause general cytotoxic effects (Figs. 1d-e**,** Additional file 1a), that have been observed by others [47, 48]. Second, the IL-12-LNP was effectively delivered to the HCC (Fig. 1c) achieving what appears to be associated with a more durable anti-tumor response. Third, we are the first to show a reduction in HCC tumor growth upon IL-12 therapy, by using dynamic MRI measurements (Figs. 2a-e).

Many studies have demonstrated that IL-12 therapy against tumors is associated with the recruitment of an immune response [47, 49–51]. We found that IL-12-LNP did not alter MYC protein levels (Fig. 3d), but did result in a robust recruitment of activated (CD44^+^, Figs. 4e-f) helper T-lymphocytes (CD3^+^ CD4^+^, Figs. 4a-d), and other CD4^+^ immune cells (Figs. 4c-d). A recent study demonstrated that IL-12 promotes HCC regression by polarizing the macrophages to the M1 phenotype [18]. Further studies need to be conducted to evaluate the phenotype and activation status of CD4^+^ immune cells other than T cells, such as macrophages and dendritic cells (DCs).

Finally, many investigators are attempting to identify drugs that target MYC for the treatment of cancer. However, we have suggested recently, that MYC-driven cancers may be generally sensitive to immune therapies, since MYC appears to play a major role in suppressing the immune response against tumors [52, 53]. Moreover, therapies that target the vulnerability of MYC-driven cancers rather than its direct suppression may avoid the toxicity that would be expected in normal tissues. Our current study demonstrates that IL-12 may be one such therapy, which if designed robustly can be delivered specifically to the tumor and can block tumor growth without damaging surrounding normal cells. Our work should inspire further efforts to develop IL-12-LNP as a therapeutic for human cancer.

## Additional files

Additional file 1: IL-12-LNP does not induce toxicity in healthy mice. (a) Evaluation of toxicity of IL-12 oligonucleotide therapy (IL-12-LNP) as measured by change in body weight of healthy mice post treatment as compared to NST-LNP-treated healthy mice (*n* = 3 mice per group). (TIFF 14745 kb)

Additional file 2: HCC clearance by IL-12-LNP is associated with anti-tumor immunological changes. (a-b) Splenic weights depicted as percentage of initial body weights for NST-LNP controls (*n* = 7), and IL-12-LNP-treated (n = 7) healthy mice (a); and for NST-LNP controls (n = 7), and IL-12-LNP-treated (n = 7) MYC-driven HCC mice (b). Data are represented as mean ± s.d. (c) Quantitative real time PCR comparing mRNA levels of IFNγ between NST-LNP (n = 7) and IL-12-LNP (n = 7) treated HCC bearing mice. Each dot represents a single mouse and is the average of three technical replicates. Data are represented as median ± interquartile range. (TIFF 14745 kb)

## Abbreviations

HCC: Hepatocellular Carcinoma
GEO: Gene Expression Omnibus
MRI: Magnetic Resonance Imaging
IL-12: Interleukin-12
MYC: v-myc avian myelocytomatosis viral oncogene homolog
OS: overall survival
IHC: immunohistochemistry
NK cell: natural killer cell
TCR: T cell receptor
IL-12R: Interleukin-12 receptor
LNP: Lipid Nanoparticle
NST: no start codon
hMYC: human MYC
mRNA: messenger RNA
GOT1: Glutamic-Oxaloacetic Transaminase 1
ALPL: Alkaline Phosphatase
GPT: Glutamic Pyruvic Transaminase
DCs: Dendritic Cells
