# Supplementary figures and images for: Lipid nanoparticles that deliver IL-12 messenger RNA suppress tumorigenesis in MYC oncogene-driven hepatocellular carcinoma
Source: J Immunother Cancer. 2018 Nov 20;6:125. doi: 10.1186/s40425-018-0431-x (PMC6247677; doi:10.1186/s40425-018-0431-x)

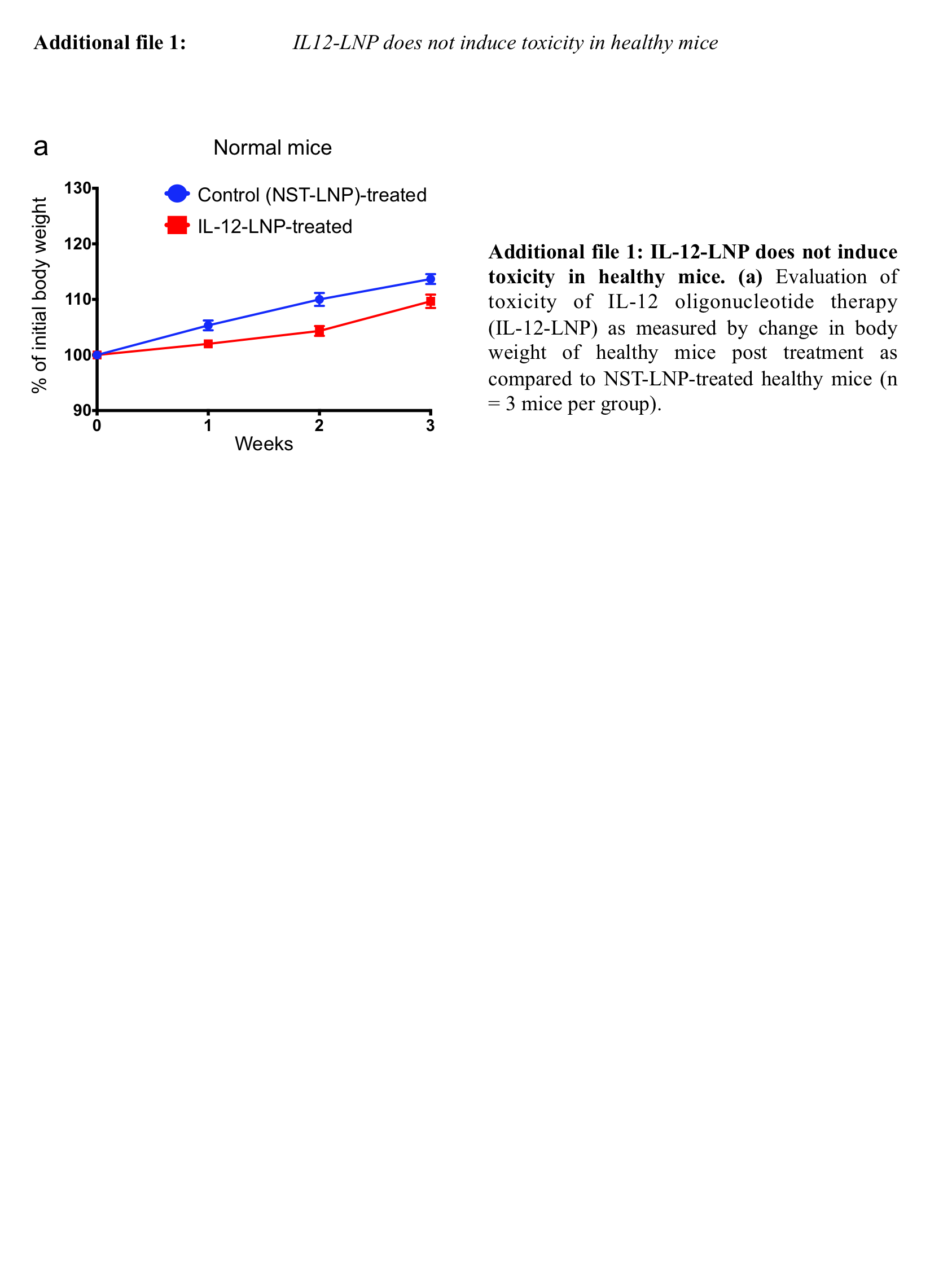

Supplement: Supplementary file 1 — IL-12-LNP does not induce toxicity in healthy mice. (a) Evaluation of toxicity of IL-12 oligonucleotide therapy (IL-12-LNP) as measured by change in body weight of healthy mice post treatment as compared to NST-LNP-treated healthy mice (n = 3 mice per group). (TIFF 14745 kb) [file 40425_2018_431_MOESM1_ESM.tiff]

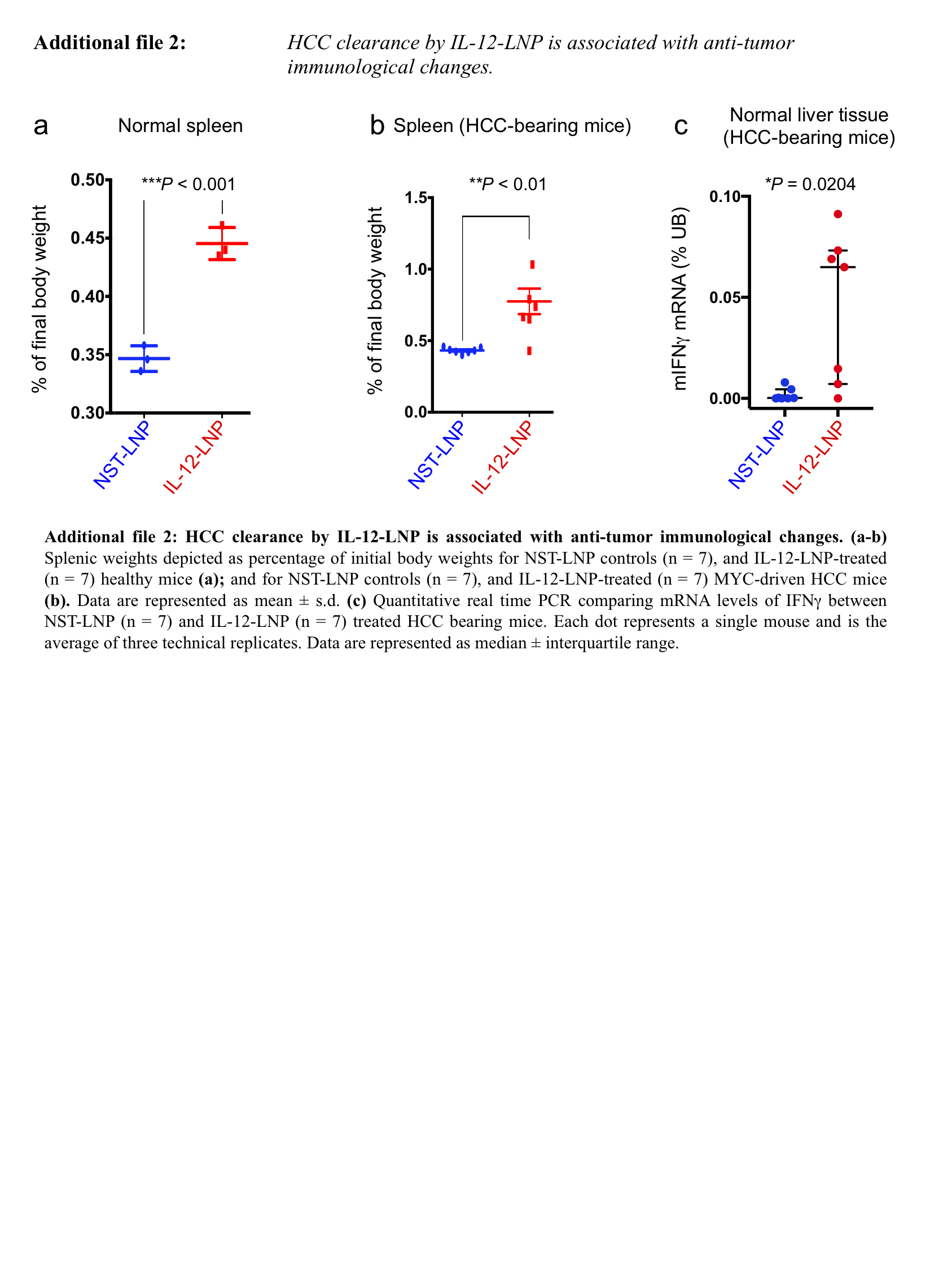

Supplement: Supplementary file 2 — HCC clearance by IL-12-LNP is associated with anti-tumor immunological changes. (a-b) Splenic weights depicted as percentage of initial body weights for NST-LNP controls (n = 7), and IL-12-LNP-treated (n = 7) healthy mice (a); and for NST-LNP controls (n = 7), and IL-12-LNP-treated (n = 7) MYC-driven HCC mice (b). Data are represented as mean ± s.d. (c) Quantitative real time PCR comparing mRNA levels of IFNγ between NST-LNP (n = 7) and IL-12-LNP (n = 7) treated HCC bearing mice. Each dot represents a single mouse and is the average of three technical replicates. Data are represented as median ± interquartile range. (TIFF 14745 kb) [file 40425_2018_431_MOESM2_ESM.tiff]
